# Supporting the development of a health benefits package in Malawi

**DOI:** 10.1136/bmjgh-2017-000607

**Published:** 2018-04-09

**Authors:** Jessica Ochalek, Paul Revill, Gerald Manthalu, Finn McGuire, Dominic Nkhoma, Alexandra Rollinger, Mark Sculpher, Karl Claxton

**Affiliations:** 1 Centre for Health Economics, University of York, York, UK; 2 Department of Planning and Policy Development, Ministry of Health, Lilongwe, Malawi; 3 Department of Global Health and Development, London School of Hygiene and Tropical Medicine, London, UK; 4 Ministry of Agriculture, Irrigation and Water Development, Lilongwe, Malawi

**Keywords:** health systems, health policy, health economics

## Abstract

Malawi, like many low-income and middle-income countries, has used health benefits packages (HBPs) to allocate scarce resources to key healthcare interventions. With no widely accepted method for their development, HBPs often promise more than can be delivered, given available resources. An analytical framework is developed to guide the design of HBPs that can identify the potential value of including and implementing different interventions. It provides a basis for informing meaningful discussions between governments, donors and other stakeholders around the trade-offs implicit in package design. Metrics of value, founded on an understanding of the health opportunity costs of the choices faced, are used to quantify the scale of the potential net health impact (net disability adjusted life years averted) or the amount of additional healthcare resources that would be required to deliver similar net health impacts with existing interventions (the financial value to the healthcare system). The framework can be applied to answer key questions around, for example: the appropriate scale of the HBP; which interventions represent ‘best buys’ and should be prioritised; where investments in scaling up interventions and health system strengthening should be made; whether the package should be expanded; costs of the conditionalities of donor funding and how objectives beyond improving population health can be considered. This is illustrated using data from Malawi. The framework was successfully applied to inform the HBP in Malawi, as a core component of the country’s Health Sector Strategic Plan II 2017–2022.

**Summary box**

**What is already known about this topic?**

- Health benefits packages (HBPs) are commonly used to set out what should be included in a publicly subsidised package of healthcare interventions to make progress towards the Sustainable Development Goal target 3.8 of Universal Health Coverage (UHC) in low-income and middle-income countries (LMICs).
- HBP design has typically failed to take proper account of all constraints faced (eg, healthcare expenditure, infrastructure and donor restrictions) and has not been informed by explicit analysis that can identify the potential value of including and implementing different interventions; as a result, HBPs are rarely fully implemented and so access to the most valuable interventions is restricted.

**What are the new findings?**

- The analytic framework is founded on an understanding of the health opportunity costs of the choices faced and so can offer a transparent, principles-based approach to informing the content and scale of a HBP with existing resources, the value of expanding the HBP and the incremental reallocation of resources within the package.
- An assessment of health opportunity costs makes it possible to report the potential net health impact (net disability adjusted life years averted) of including a particular intervention or the amount of additional healthcare resources that would be required to deliver similar net health impacts (financial value to the healthcare system).
- This enables interventions that should be prioritised to be identified and the value of implementation efforts and health system strengthening to be assessed and also indicates the value of expanding the package, the costs of the conditionalities of donor funding and the trade-offs required when considering other objectives.

**Summary box**

**What are the recommendations for policy and practice?**

- The purpose of this analytic framework is not to prescribe a particular package or what health expenditure ought to be, rather it shows how evidence, such as it is, can be marshalled and analysis presented in a way that can empower Ministries of Health (MoH) as they engage with a range of stakeholders in making explicit, accountable and evidence-based decisions.
- The framework can contribute to advancing UHC goals in a way that makes best use of the resources available and shows the value of committing additional resources for healthcare, addressing common challenges and trade-offs faced by diverse healthcare systems in LMICs.
- The successful application by the Malawian MoH in developing Malawi’s Health Sector Strategic Plan II (2017–2022) demonstrates its practicality in making best use of often-limited evidence in a low-income country setting.

## Introduction

Sustainable Development Goal target 3.8 is to ‘Achieve universal health coverage, including financial risk protection, access to quality essential healthcare services and access to safe, effective, quality and affordable essential medicines and vaccines for all’ by 2030.^1^ However, the resources available for healthcare are limited, so not all services can be provided. Health benefits packages (HBPs) are an increasingly common way of explicitly defining which health services are provided through public expenditure as progress is made towards Universal Health Coverage (UHC).^2–4^ At least 64 low-income and middle-income countries (LMICs) defined some form of HBP by 2012.^4 5^ However, packages vary widely in terms of how benefits are defined, the cost of the packages, the coverage levels actually achieved and the methods used to inform their design.^3 4^

Despite the frequent and increasing use of HBPs in LMICs, package design often suffers from a number of common flaws. The process of benefits package design is often non-transparent, non-inclusive and not informed by explicit analysis that makes best use of the often-limited evidence available. Decisions can, therefore, appear ad hoc rather than evidence-based. In particular, analysis rarely reflects the impact of various constraints on intervention provision and uptake. Therefore, the health opportunity cost of decisions is seldom accounted for. These issues are highly context-specific and ultimately affect the scale of the additional benefits and costs of including particular interventions. Attempts have been made to address some of the evidential shortcomings with ‘global public goods’ (eg, the DCP series). However, they often fail to address local conditions such as constraints on provision and uptake. As a result, packages generally promise more than they can deliver and healthcare is implicitly rationed with the most essential care not necessarily being delivered.^3^ If HBPs are to advance UHC goals in a way that makes best use of the resources available for healthcare and informs how additional resources can most productively and equitably be used, an analytic framework is required that exposes the inevitable trade-offs to assist decision makers in their design.^5^

Such a framework was developed in response to a request by the Ministry of Health of Malawi to researchers at the Centre for Health Economics, University of York for an analytic framework to guide resource allocation within the Health Sector Strategic Plan 2017–2022 (HSSP II). The framework needed to inform key questions posed by the Ministry of Health:

- What is the appropriate scale of the HBP?
- Which interventions represent ‘best buys’ for the healthcare system (HCS) and should be prioritised?
- Where should investments in scaling up interventions and health system strengthening be made?
- Should the package be expanded to include additional interventions?
- What are the costs of the conditionalities of donor funding?
- How can objectives beyond improving population health be considered?

The remainder of this paper is organised as follows. The health policy context in Malawi is initially introduced. Then the framework for designing HBPs is described, and an illustrative analysis is presented to answer each of the questions posed by applying the framework to data from Malawi. The application of the framework by the Malawian government to the development of a HBP for the HSSP-II is described, before the applicability of the framework to other settings and suggestions for future work are discussed.

## Health policy context in Malawi

Malawi introduced its first essential health package (EHP) in 2004 as a means of allocating collectively pooled resources for healthcare in conjunction with the initiation of a health Sector-Wide Approach (SWAp) to funding and resource allocation.^6^ As part of the SWAp, donors provided general budget support and resource allocation decisions were made centrally by Government.^7^ This replaced a fragmented vertical disease-based approach to funding.^8^ The donor share of funding for the SWAp gradually increased from 30% in 2004/2005 to 56% in 2006/2007.^9^ However, following the ‘cashgate’ scandal of 2013, many donors moved away from general budget support.^10 11^ In the 2014/2015 financial year, donors contributed only 8% of SWAp pool resources, while the remaining 92% (MK65.8 billion) were raised domestically,^12^ with donors instead returning to vertical disease-based funding channels. In FY 2015/2016, on-budget funding (ie, government-raised funds and direct budget support from donors) made up only 32% of total funding while the remainder was mostly off-budget discrete project support.^12 13^

Despite the changing fiscal and political landscape, Malawi has continued to use HBPs to prioritise spending from both government and donor partners in the health sector. However, its first two HBPs in 2004 and 2011^6 14^ were unsustainable, estimated to cost between 83% and 182% of total health expenditure, of which the package forms only a part.^6 14–16^ As is common with packages globally, the HBPs could not be implemented resulting in inequitable variations in access to care and in many circumstances priority ‘best buy’ interventions were not available.^17^

## A framework for designing HBPs

To address the policy questions in Malawi, a general framework was required that enabled the quantification of the health gains that would result from different potential HBPs (ie, with different choices of interventions) and account for actual constraints on implementation, donor restrictions and objectives other than health improvement. Including an intervention in the HBP commits resources that could otherwise have funded other interventions that also improve health. These forgone interventions and their associated health improvements represent the health opportunity cost of including a particular intervention in the HBP.

An explicit and evidence-based assessment of health opportunity costs enables metrics of value to be reported. These indicate the scale of the potential health impact of including an intervention in the HBP net of associated health opportunity costs and of ensuring it is fully implemented. This information can be reported in health or monetary terms, which in turn can inform the value of committing resources to implementation efforts. These metrics of value (see box 1) inform prioritisation decisions more directly than other measures that have been used previously. For example, estimates of burden of disease or cost-effectiveness ratios do not indicate the scale of population health benefits offered by providing interventions to defined populations.^18 19^

Box 1 **Metrics of value**

- Net disability adjusted life years (DALYs) averted represent the net health impact of an intervention on population health. It is the difference between the DALYs averted by an intervention and DALYs that could have been averted if the money required to deliver it had been spent on other interventions. If the intervention saves resources, it is the DALYs averted by the intervention plus the DALYs that can also be averted by including other interventions with the cost savings offered.
- The financial value to the healthcare system (the value of the intervention expressed in monetary terms) is the amount of additional healthcare resources that would be required to deliver the equivalent net DALYs averted with other interventions.

The illustrative analysis that follows relies on an empirical estimate of health opportunity costs (see box 2) and uses estimates of the costs and health effects of interventions from the *Tufts Global Health Cost-Effectiveness Registry* and WHO CHOosing Interventions that are Cost-Effective (WHO-CHOICE) analyses. Budgetary analysis to determine the total cost of the package uses drug and supply costs from a 2014 costing mid-term review of the previous HSSP made available by in-country partners Palladium and the Clinton Health Access Initiative (CHAI). The size of eligible patient populations for each intervention and an assessment of the levels to which interventions were actually implemented in Malawi in 2014 use bottleneck analysis and data from CHAI. Therefore, the data requirements do not extend far beyond the data collected regularly in many LMIC health systems. Shortfalls in data availability are inevitable, in any environment, but the framework allows the best use to be made of routinely collected local data, which complements relevant and available globally available data, within decision-making processes.

Box 2 **Estimating health opportunity costs**

- Recent research, although in high-income countries, has demonstrated that an empirical assessment of health opportunity costs is possible based on estimates of the health effects of changes in healthcare expenditure.^28–30^
- Some of these estimates have been used to infer possible health opportunity costs in low-income and middle-income countries (LMICs).^31^
- Published estimates of the effect of changes in health expenditure on mortality using country-level data, including LMICs, can also be used to estimate health opportunity costs (cost per disability adjusted life year (DALY) averted) for particular healthcare system, reflecting their demography, epidemiology, healthcare expenditure, income and other characteristics.^32^
- The results of this type of empirical estimation suggest that the GDP per capita-based ‘thresholds’ that have been widely used to judge cost-effectiveness in LMICs are likely to be significantly higher than an assessment of health opportunity costs.
- In Malawi, the range of estimates available suggests that $61 spent on healthcare at the margin would be expected to avert one DALY.^31 32^

## Informing key questions in HBP design

### What is the appropriate scale of the HBP?

**Figure 1:**
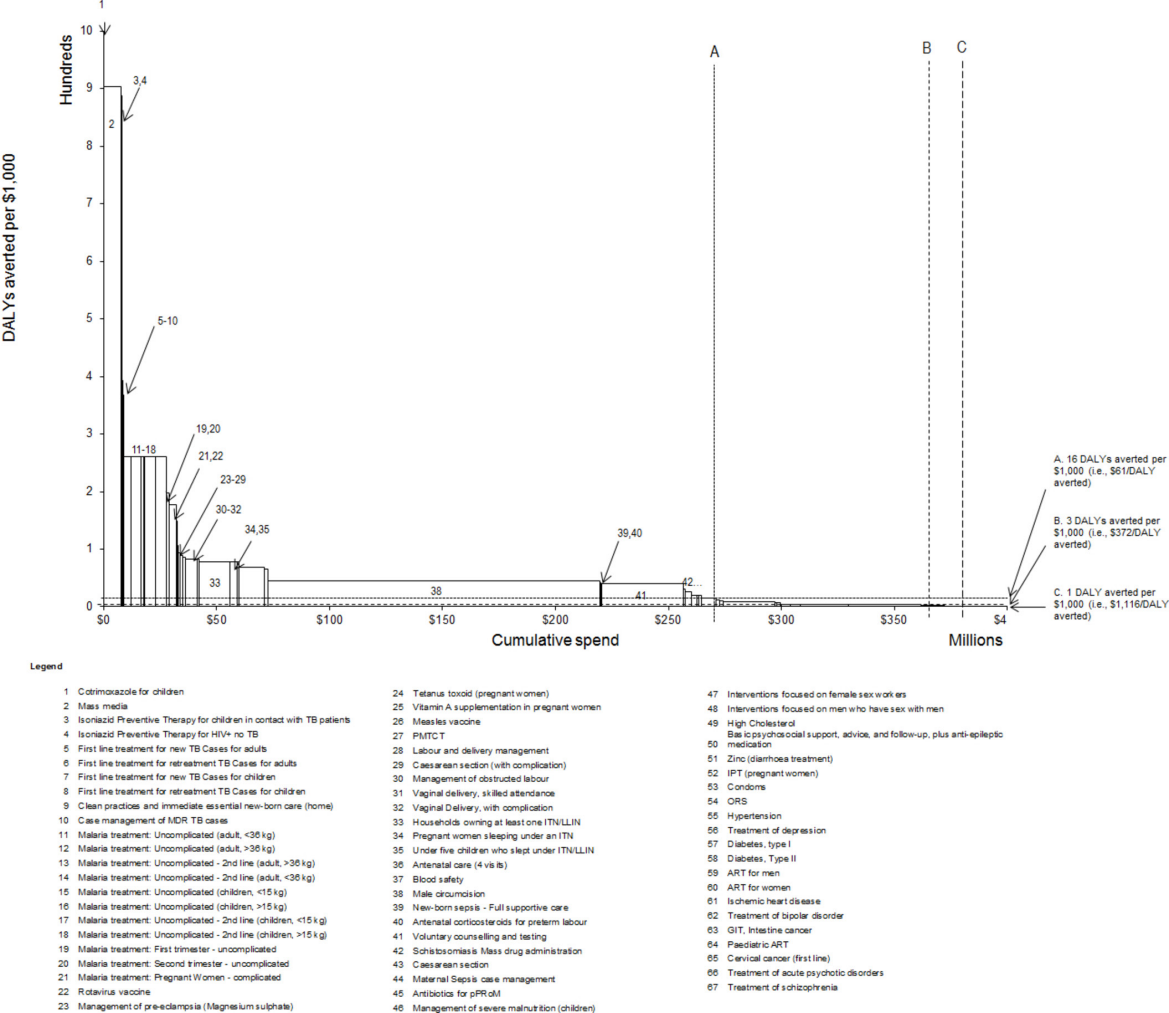
DALYs averted per $1000 and different budgets. ART, antiretroviral therapy; DALY, disability adjusted life year; GIT, gastrointestinal; ITN, insecticide-treated bed net; IPT, intermittent preventive therapy; LLIN, long-lasting insecticidal net; ORS, oral rehydration salts; PMTCT, prevention of mother to child transmission; pPRoM, preterm premature rupture of membranes.

Figure 1 shows the interventions for which all required estimates were available, ordered and numbered from the lowest (left) to highest (right) ratio of cost per disability adjusted life year (DALY) averted. The height of each bar represents the intervention’s effectiveness-cost ratio, and the width of each bar represents the intervention’s total cost.^20^ The latter is a function of the number of patients that require it and the cost per patient of delivering it, assuming each intervention is fully implemented. If Malawi can currently afford to pay up to $61 to avert one DALY (ie, 16 DALYs averted per $1000, see box 2), interventions 1–48 would be included in the HBP resulting in a budget of $265 million (shown as vertical dashed line A).

The estimate of $61 per DALY might be regarded as too low if policy makers felt able to commit more funding to healthcare given the size of other budgets and overall public resources. Higher estimates imply an expanded EHP with a larger budget. Once the health that is likely to be delivered by greater healthcare expenditure is set out, it then becomes possible to have a more meaningful deliberation about how Malawi’s public finance resources might be allocated between competing claims (health education, infrastructure and so on) and/or how increases in public finance to accommodate increased health expenditure might be achieved.

For example, if the widely cited norms of 1 or 3 GDP per capita are adopted, the ‘threshold’ would be $372 or $1116 per DALY averted, with interventions 1–60 or 1–65 included, implying a budget of $362 or $380 million, respectively, as indicated by dashed lines B and C.^21^ It should be noted that in this illustrative example only those interventions where estimates of cost, health benefit, eligible population and level of implementation were available were included. Since other possible interventions are missing from figure 1, the difference in total budget for increases in the ‘threshold’ will tend to be underestimated, especially if high-cost interventions are under-represented. This also illustrates the experience of previous EHPs in Malawi and many other LMICs, where adopting ‘threshold’ norms that exceed the reality of health opportunity costs results in the inclusion of more in the package than can actually be funded. This leads to arbitrary and inequitable rationing, reduced health impact of the more limited resources that are actually available.^5^

### Which interventions represent ‘best buys’ for the healthcare system and should be prioritised?

While figure 1 provides a useful way to visualise the budget implications of using a higher or lower ‘threshold’ value, cost per DALY averted ratios are not useful for prioritising interventions because they do not indicate the scale of the potential health impact. Table 1 ranks interventions according to the net DALYs averted that they achieve, again initially assuming they are fully implemented.

**Table 1:** Prioritising interventions in terms of impact on overall population health (net DALYs averted)

Intervention	(1)*	(2)†	(3)‡	(4)§	(5)¶	(6)**	(7)††	(8)‡‡
	ICER rank (most to least cost-effective)	ICER ($)	Population DALYs averted per 1000	Cases per annum (1000 s)	Total cost ($1000 s)	Cumulative cost ($1000 s)	Total DALYs averted	Net DALYs averted (1000s)
Male circumcision	38	22	45	4073	146 730	146 730	39 634	25 423
Management of obstructed labour	30	12	86	92	1100	147 829	2497	2026
Isoniazid preventive therapy for HIV+ no TB	4	1	887	55	80	147 909	1118	1098
First-line treatment for new TB cases for adults	5	3	393	14	178	148 087	1045	1002
First-line treatment for new TB cases for children	7	3	393	12	117	148 204	888	851
Management of pre-eclampsia (magnesium sulfate)	23	6	168	20	45	148 249	535	483
Clean practices and immediate essential newborn care (home)	9	3	368	671	416	148 665	237	227
Households owning at least one ITN/LLIN	33	13	77	6752	13 737	162 402	228	180
Caesarean section	43	32	31	34	672	163 073	327	157
Mass media	2	1	903	16 879	7609	170 682	150	148
Labour and delivery management	28	11	89	918	1281	171 964	170	139
PMTCT of HIV	27	11	94	53	600	172 564	157	130
First-line treatment for retreatment TB cases for adults	6	3	393	2	100	172 664	131	125
Caesarean section (with complication)	29	12	86	5	172	172 836	137	111
First-line treatment for retreatment TB cases for children	8	3	393	2	66	172 901	111	106
Malaria treatment: first trimester— uncomplicated	19	5	198	305	1025	173 927	109	100
Malaria treatment: Second trimester—uncomplicated	20	5	198	305	235	174 162	109	100
Voluntary counselling and testing	41	25	40	8031	36 309	210 471	167	98
Tetanus toxoid (pregnant women)	24	7	149	918	115	210 585	104	92
Measles vaccine	26	9	106	651	528	211 113	107	90
Rotavirus vaccine	22	6	177	651	3097	214 210	88	80
Antenatal care (four visits)	36	15	68	918	11 230	225 440	90	68
Malaria treatment: uncomplicated (adult, <36 kg)	11	4	260	4372	3463	228 903	59	56
Malaria treatment: uncomplicated (adult, >36 kg)	12	4	260	4372	4267	233 170	59	56
Malaria treatment: uncomplicated—second line (adult, >36 kg)	13	4	260	4372	1186	234 356	59	56
Malaria treatment: uncomplicated—second line (adult, <36 kg)	14	4	260	4372	593	234 949	59	56
Vaginal delivery, skilled attendance	31	12	83	918	5181	240 130	67	54
Isoniazid preventive therapy for children in contact with patients with TB	3	1	900	2	7	240 138	45	44
Interventions focused on men who have sex with men	48	51	20	34	1256	241 393	232	40
Pregnant women sleeping under an ITN	34	13	77	1469	2990	244 383	50	39
Newborn sepsis—full supportive care	39	24	42	81	417	244 800	60	37
Management of severe malnutrition (children)	46	50	20	51	2437	247 237	199	36
Vitamin A supplementation in pregnant women	25	7	140	124	125	247 362	33	30
Antenatal corticosteroids for preterm labour	40	25	40	165	406	247 768	47	28
Interventions focused on female sex workers	47	51	20	23	655	248 423	161	28
Cotrimoxazole for children	1	cost saving		127	220	248 643	0	23
Malaria treatment: uncomplicated (children, <15 kg)	15	4	260	1042	4576	253 219	14	13
Malaria treatment: uncomplicated (children, >15 kg)	16	4	260	1042	4768	257 987	14	13
Malaria treatment: uncomplicated—second line (children, <15 kg)	17	4	260	1042	35	2 58 023	14	13
Malaria treatment: uncomplicated—second line (children, >15 kg)	18	4	260	1042	71	258 093	14	13
Under five children who slept under ITN/LLIN	35	13	77	494	1006	259 099	17	13
Schistosomiasis mass drug administration	42	29	35	389	77	259 176	24	13
Antibiotics for pPRoM	45	40	25	64	39	259 214	30	10
Blood safety	37	15	66	40	1626	260 840	12	9
Vaginal delivery, with complication	32	12	83	138	804	261 644	10	8
Maternal sepsis case management	44	39	26	64	2731	264 375	20	7
Malaria treatment: pregnant Women —complicated	21	5	198	16	140	264 515	6	5
Case management of MDR TB cases	10	3	297	0	12	264 527	5	5
GIT tract cancer	63	804	1	0	3	264 530	0	0
Cervical cancer (first line)	65	1087	1	2	162	264 691	0	0
Ischaemic heart disease	61	453	2	128	4	264 695	0	0
IPT of malaria (pregnant women)	52	110	9	735	35	264 730	0	0
Diabetes, type I	57	296	3	23	4304	269 034	0	0
High cholesterol	49	68	15	223	6703	275 737	1	0
Basic psychosocial support, advice and follow-up, plus antiepileptic medication	50	82	12	506	1266	277 003	1	0
Treatment of depression	56	265	4	169	332	277 334	0	0
Diabetes, Type II	58	296	3	138	4211	281 545	0	-1
Treatment of acute psychotic disorders	66	1646	1	169	958	282 503	0	-1
Treatment of bipolar disorder	62	557	2	523	10 362	292 865	0	-1
Treatment of schizophrenia	67	1646	1	2363	13 413	306 278	0	−10
Hypertension	55	159	6	846	1338	307 616	44	−71
Zinc (diarrhoea treatment)	51	99	10	7455	1788	309 404	244	−150
ORS	54	153	7	8662	937	310 341	147	−221
Condoms	53	127	8	8031	22 883	333 223	482	−517
ART for men	59	312	3	332	21 159	354 382	1005	−4104
ART for women	60	312	3	509	32 440	386 823	1541	−6292
Paediatric ART	64	892	1	107	7657	394 480	1556	−21 074

*ICER rank: from most to least cost-effective.

†ICER ($): translated to 2016 US$ from original sources.

‡Population DALYs averted per 1000: 1/cost effectiveness ratio × 1000.

§Cases per annum (1000s): size of eligible patient populations for each intervention.

¶Total cost ($1000s): cost per patient × number of patients requiring the intervention.

**Cumulative costs ($1000 s): the total cost of the intervention and all previous interventions.

††Total DALYs averted: DALYs averted per patient × number of patients requiring the intervention.

‡‡Net DALYs averted (1000 s): Difference between the DALYs averted by an intervention and DALYs that could have been averted with any additional HCS resources required to implement it, calculated as DALYs averted per patient × number of patients requiring the intervention – cost per patient × number of patients requiring the intervention/(1/estimated marginal productivity of the HCS).

ART, antiretroviral therapy; DALY, disability adjusted life year; GIT, gastrointestinal; HCS, healthcare system; ICER, incremental cost effectiveness ratio; IPT, intermittent preventive therapy; ITN, insecticide-treated bed net; LLIN, long-lasting insecticidal net; MDR, multidrug resistant; ORS, oral rehydration salts; PMTCT, prevention of mother to child transmission; pPRoM, preterm premature rupture of membranes; TB, tuberculosis.

Ranking interventions by the net DALYs they avert results in a different ordering than ranking by ratios because the net DALYs averted reflects the size of the patient population as well as the individual health effect and costs. For example, management of obstructed labour, which is ranked 30th (ie, intervention 30) by cost-effectiveness ratios, is ranked second by net benefit because it generates a large health impact and remains higher than other interventions even when health opportunity costs are considered. The intervention ranked first by cost-effectiveness ratios (ie, intervention 1—cotrimoxazole prophylaxis for children) averts fewer net DALYs than other interventions that impose costs on the system, despite being cost saving.

Those interventions to the right of the dashed line ‘A’ in figure 1 would result in negative overall population health impacts (ie, negative net DALYs averted) if they had been included in the package, as shown in table 1. This is because the cost associated with those interventions could be used elsewhere to better effect (ie, the resources would generate higher DALYs averted than if used for these particular interventions).

Interventions that represent ‘best buys’ for the HCS and should be prioritised are those that generate the most net health. These include HIV prevention strategies (including prevention, testing and treatment strategies); treatment for tuberculosis; maternal and child health interventions (such as management of pre-eclampsia, caesarean section and labour and delivery management) and prevention of and treatment for malaria.

### Where should investments in scaling up interventions and health system strengthening be made?

In Malawi, the mean actual implementation level in 2014 among all interventions included in the analysis is 46%, with a range of 1%–100%. Constraints to implementation include, on the demand side, individuals’ lack of perceived benefits of care and difficulty in getting to clinics due to poor road infrastructure and, on the supply side, lack of equipment, lack of trained staff, supply chain bottlenecks, lack of beds, water and electricity shortages.^22^ As a result, less money is spent delivering interventions and fewer DALYs are averted (see Columns 7 and 9 in table 2, respectively.) This results in a gap between current and potential spend of $198 million. One possible way of investing this spending gap is on policies to improve implementation levels, for specific interventions or across the HCS. Which interventions to invest in depends on the health gains that could be achieved by such investments. Table 2 ranks interventions by financial value to the HCS (Column 12).

**Table 2:** Net DALYs averted at full and actual implementation levels

Intervention	(1)	(2)	(3)	(4)	(5)	(6)	(7)	(8)	(9)	(10)	(11)	(12)
	ICER ranking	ICER ($)	Pop. DALYs averted per 1000	Cases per annum (1000 s)	Actual imp. level	Total cost (full imp.; $1000s)	Total cost (actual imp.; $1000s)	Total DALYs averted (full imp.; 1000s)	Total DALYs averted (actual imp.; 1000s)	Net DALYs averted (full imp.; 1000s)	Net DALYs averted actual imp.; 1000s)	Financial value to the healthcare system of moving from actual to full imp. ($1000s)
Male circumcision	38	22	45	4073	12%	146 730	17 608	39 634	4756	25 423	3051	1 372 314
Isoniazid preventive therapy for HIV+ no (TB)	4	1	887	55	50%	80	40	1118	559	1098	549	33 673
First-line treatment for new TB cases for adults	5	3	393	14	64%	178	114	1045	669	1002	641	22 122
First-line treatment for new TB cases for children	7	3	393	12	64%	117	75	888	568	851	545	18 789
Clean practices and immediate essential newborn care (home)	9	3	368	671	0%	416	–	237	–	227	–	13 909
Management of pre-eclampsia (magnesium sulfate)	23	6	168	20	80%	45	36	535	428	483	386	5923
Voluntary counselling and testing	41	25	40	8031	15%	36 309	5446	167	25	98	15	5120
Rotavirus vaccine	22	6	177	651	0%	3097	–	88	–	80	–	4920
Households owning at least one ITN/LLIN	33	13	77	6752	56%	13 737	7706	228	128	180	101	4847
Malaria treatment: first trimester—uncomplicated	19	5	198	305	33%	1025	341	109	36	100	33	4087
PMTCT	27	11	94	53	55%	600	332	157	87	130	72	3561
Malaria treatment: uncomplicated—second line (adult, >36 kg)	13	4	260	4372	2%	1186	18	59	1	56	1	3354
Malaria treatment: uncomplicated—second line (adult, <36 kg)	14	4	260	4372	4%	593	21	59	2	56	2	3285
Labour and delivery management	28	11	89	918	65%	1281	833	170	111	139	91	2992
First-line treatment for retreatment TB cases for adults	6	3	393	2	65%	100	65	131	85	125	81	2688
Mass media	2	1	903	16 879	71%	7609	5402	150	107	148	105	2627
Malaria treatment: uncomplicated (adult, <36 kg)	11	4	260	4372	30%	3463	1039	59	18	56	17	2383
Interventions focused on men who have sex with men	48	51	20	34	5%	1256	63	232	12	40	2	2311
Antenatal care (four visits)	36	15	68	918	46%	11 230	5110	90	41	68	31	2289
First-line treatment for retreatment TB cases for children	8	3	393	2	65%	66	43	111	72	106	69	2283
Malaria treatment: second trimester—uncomplicated	20	5	198	305	67%	235	157	109	72	100	67	2047
Antenatal corticosteroids for preterm labour	40	25	40	165	0%	406	–	47	–	28	–	1718
Newborn sepsis— full supportive care	39	24	42	81	40%	417	167	60	24	37	15	1346
Cotrimoxazole for children	1	cost saving		127	13%	220	28	0	0	23	3	1208
Interventions focused on female sex workers	47	51	20	23	30%	655	197	161	48	28	8	1184
Vaginal delivery, skilled attendance	31	12	83	918	65%	5181	3368	67	43	54	35	1153
Malaria treatment: uncomplicated (adult, >36 kg)	12	4	260	4372	70%	4267	2987	59	41	56	39	1021
Tetanus toxoid (pregnant women)	24	7	149	918	84%	115	96	104	87	92	77	905
Malaria treatment: uncomplicated—second line (children, <15 kg)	17	4	260	1042	2%	35	1	14	0	13	0	799
Malaria treatment: uncomplicated—second line (children,>15 kg)	18	4	260	1042	4%	71	2	14	0	13	0	783
Schistosomiasis mass drug administration	42	29	35	389	13%	77	10	24	3	13	2	670
Vitamin A supplementation in pregnant women	25	7	140	124	65%	125	81	33	22	30	19	634
Malaria treatment: uncomplicated (children, >15 kg)	16	4	260	1042	40%	4768	1907	14	6	13	5	487
Antibiotics for pPRoM	45	40	25	64	30%	39	12	30	9	10	3	450
Maternal sepsis case management	44	39	26	64	0%	2731	–	20	–	7	–	449
Management of severe malnutrition (children)	46	50	20	51	80%	2437	1949	199	159	36	29	446
Isoniazid preventive therapy for children in contact with patients with TB	3	1	900	2	85%	7	6	45	38	44	38	408
Malaria treatment: uncomplicated (children, <15 kg)	15	4	260	1042	60%	4576	2746	14	8	13	8	325
Vaginal delivery, with complication	32	12	83	138	51%	804	410	10	5	8	4	242
Measles vaccine	26	9	106	651	99%	528	523	107	106	90	89	55
Management of obstructed labour	30	12	86	92	100%	1100	1100	2497	2497	2026	2026	–
Caesarean section	43	32	31	34	100%	672	672	327	327	157	157	–
Caesarean section (with complication)	29	12	86	5	100%	172	172	137	137	111	111	–
Pregnant women sleeping under an ITN	34	13	77	1469	100%	2990	2990	50	50	39	39	–
Under five children who slept under ITN/LLIN	35	13	77	494	100%	1006	1006	17	17	13	13	–
Blood safety	37	15	66	40	100%	1626	1626	12	12	9	9	–
Malaria treatment: pregnant women— complicated	21	5	198	16	100%	140	140	6	6	5	5	–
Case management of MDR TB cases	10	3	297	0	100%	12	12	5	5	5	5	–
Malaria treatment: pregnant women—complicated	21	5	198	16	100%	140	140	6	6	5	5	–
Caesarean section (with complication)	29	12	86	5	100%	172	172	137	137	111	111	–
Management of obstructed labour	30	12	86	92	100%	1100	1100	2497	2497	2026	2026	–
Pregnant women sleeping under an ITN	34	13	77	1469	100%	2990	2990	50	50	39	39	–
Under five children who slept under ITN/LLIN	35	13	77	494	100%	1006	1006	17	17	13	13	–
Blood safety	37	15	66	40	100%	1626	1626	12	12	9	9	–
Caesarean section	43	32	31	34	100%	672	672	327	327	157	157	–
IPT (pregnant women)	52	110	9	735	100%	35	35	0	0	−0	−0	–
GIT cancer	63	804	1	0	50%	3	1	0	0	−0	−0	−0
Cervical cancer (first line)	65	1087	1	2	50%	162	81	0	0	−0	−0	−0
Ischaemic heart disease	61	453	2	128	15%	4	1	0	0	−0	−0	−2
Diabetes, type I	57	296	3	23	15%	4304	646	0	0	−0	−0	−5
High cholesterol	49	68	15	223	1%	6703	67	1	0	−0	−0	−6
Basic psychosocial support, advice and follow-up, plus antiepileptic medication	50	82	12	506	3%	1266	38	1	0	−0	−0	−14
Treatment of depression	56	265	4	169	1%	332	3	0	0	−0	−0	−23
Diabetes, Type II	58	296	3	138	15%	4211	632	0	0	−1	−0	−30
Treatment of acute psychotic disorders	66	1646	1	169	1%	958	10	0	0	−1	−0	−42
Treatment of bipolar disorder	62	557	2	523	3%	10 362	321	0	0	−1	−0	−87
Treatment of schizophrenia	67	1646	1	2363	14%	13 413	1878	0	0	−10	−1	−512
Hypertension	55	159	6	846	10%	1338	134	44	4	−71	−7	−3912
ORS	54	153	7	8662	69%	937	647	147	102	−221	−152	−4197
Zinc (diarrhoea treatment)	51	99	10	7455	0%	1788	–	244	–	−150	–	−9207
Condoms	53	127	8	8031	47%	22 883	10 755	482	226	−517	−243	−16 813
ART for men	59	312	3	332	75%	21 159	15 961	1005	758	−4104	−3,096	−61 848
ART for women	60	312	3	509	82%	32 440	26 669	1541	1267	−6292	−5,173	−68 665
Paediatric ART	64	892	1	107	25%	7657	1892	1556	384	−21 074	−5,206	−973 334

ART, antiretroviral therapy; DALY, disability adjusted life year; GIT, gastrointestinal; ICER, incremental cost effectiveness ratio; IPT, intermittent preventive therapy; ITN, insecticide-treated bed net; LLIN, long-lasting insecticidal net; MDR, multidrug resistant; ORS, oral rehydration salts; PMTCT, prevention of mother to child transmission; pPRoM, preterm premature rupture of membranes; TB, tuberculosis.

For example, schistosomiasis mass drug administration is only available to 13% of the eligible patient population. If it were fully implemented, it would avert 23 754 DALYs (vs only 3088 at actual implementation levels). Table 3 presents the calculations underlying the values reported in table 2. Using the $61 per DALY averted estimate of health opportunity costs, if fully implemented, schistosomiasis mass drug administration would have a net effect of 12 562 DALYs averted (vs 1633 at actual implementation). As such, scaling up from actual levels of implementation to 100% would result in an additional 10 929 net DALYs averted (the difference between net DALYs averted at full and actual implementation (Column 3), equivalent to a $670 393 value to the HCS (Column 4). This means that, at most, $670 393 could be spent on removing the constraints to implementing schistosomiasis mass drug administration for that to remain a cost-effective use of resources.

**Table 3:** Valuing scaleup: schistosomiasis mass drug administration

	Total DALYs averted	Total cost ($)	Net DALYs averted	Financial value to the healthcare system ($)
Full implementation	23 754	76 527	12 562	770 567
Actual implementation	3088	9949	1633	100 174
Value of moving from actual to full implementation	20 666	66 578	10 929	670 393

DALY, disability adjusted life year.

Aggregating the total DALYs averted at 100% implementation across the interventions in the package (49.5 million) and subtracting the total DALYs averted at actual implementation (11.4 million) gives the maximum health gains that system strengthening could achieve (38.0 million DALYs averted). This suggests that there are potentially substantial gains from investing in policies which reduce or remove constraints to implementation at the intervention level and across the HCS as a whole.

### Should the package be expanded to include additional interventions?

The Ministry of Health could accept existing constraints and instead use the budget spending gap resulting from constraints on full implementation to fund the inclusion of additional interventions not included in the initial package (ie, any intervention with cost per DALY averted estimates greater than $61). Whether this should be judged as a good use of money depends on the DALYs that can be averted by the additional interventions at actual implementation levels.

Using the spending gap ($198 million) to include interventions 49–67 would avert 2.7 million additional DALYs, resulting in a total of 14.2 million DALYs averted. This is 35.3 million fewer DALYs than could potentially be averted by investing in policies to improve implementation of already included interventions. This suggests that investing in implementation efforts should be prioritised if there are effective ways to relax the constraints. Although the effectiveness of such policies is often unknown, understanding the scale of the potential benefits can support informed judgements by decision makers. For example, even if only 14% of the potential health gains of implementation efforts were achieved using the spending gap it would be preferable to package expansion with that money. Furthermore, expanding the package may adversely impact the implementation of higher priority interventions so the additional DALYs that could be averted by including additional interventions probably overestimates the health benefits of expanding the package.

### What are the costs of the conditionalities of donor funding?

Donors, who fund approximately 70% of the HCS in Malawi, may also impose constraints through their funding arrangements.^23^ Analyses comparing the health benefits of the donor’s offers of assistance with the health opportunity cost can inform a discussion with donors about the need to impose constraints on their funding and can engage stakeholders in understanding the implications of particular policy options regarding donor offers. Such options may include accepting the donor proposal but being clear about the health opportunity cost of doing so or rejecting offers of matched funding for interventions that do not offer net health benefits. Proposals that might make the implementation of high priority interventions more difficult might be mitigated by other policies (eg, use of user fees to deter uptake of the imposed intervention). The framework provides estimates of the health opportunity cost of the constraints that a donor proposal might impose, which provide a valuable basis for explaining decisions to stakeholders.

When an intervention that is not cost-effective is included it always reduces the total health generated by the package. The difference in the health gains associated with a health maximising package that uses all available resources, including those provided by donors, and a package where the donor specifies that particular interventions be included as a condition of the resources provided, indicates the minimum health opportunity cost of these restrictions. For example, requiring that first-line treatment for cervical cancer (intervention #65 in figure 1) is included in the package as a condition of existing levels of assistance will not increase the budget, so the health opportunity cost of this requirement is the health that would have been gained by the interventions that must be removed to accommodate it. The health opportunity cost of these types of conditions will be higher if it is not the least cost-effective interventions that are displaced and/or if they make other higher priority interventions more difficult to implement. Other examples of how different types of restrictions on assistance can be assessed are illustrated in online supplementary file 1. Evidence of the scale of the health opportunity costs associated with restrictions and conditions on donor assistance enables a more informed and accountable negotiation between stakeholders including careful examination of the reasons for restrictions.

10.1136/bmjgh-2017-000607.supp1 Supplementary data

### How can objectives beyond improving population health be considered?

Inevitably, the Ministry of Health and stakeholders may want to consider a range of objectives in addition to gains in population health when making decisions about what interventions to include in the EHP. These might include, for example, using interventions to promote financial protection or to reduce health inequalities and recognising the impact of interventions on wider social objectives such as productivity. In principle, it is possible to extend the measures of benefit and opportunity cost to include these other considerations.^24 25^ In practice, this may be challenging based on available evidence, in which case it is possible to inform decisions about relevant trade-offs based on changes in population health.^26^ The health losses associated with including an intervention that would not be included on the basis of net benefit alone can be quantified in the same way as the health losses associated with conditions on donor funding. These can be weighed against the gains in other objectives that result from the inclusion of the intervention. This quantification provides policy makers with a basis to understand whether the trade-offs are worth making and a means of communicating their ultimate decisions to stakeholders.

## Application of the framework to the development of a revised EHP in Malawi

This analysis is intended to provide an analytic framework which can be used to support rather than prescribe decisions. The framework and data supporting the initial analysis were shared with the Ministry of Health in Malawi, which mandated an already existing EHP Technical Working Group (TWG) to conduct the EHP revision process (including the Ministry of Health Heads of Departments and Programme Managers, technical partners such as the local WHO office, donors, academic institutions and other key national health stakeholders). The TWG added other criteria to health maximisation including: equity (whether an intervention targeted at risk or marginalised groups); continuum of care (where interventions are linked, eg, screening and treating); complementarities (whether interventions are part of package) and exceptional donor funded interventions (donor funding for interventions that were expected to remain largely stable in the medium term). The framework was used to quantify the health gains that would result from different choices of interventions that met the agreed criteria to varying degrees, enabling explicit consideration of the necessary trade-offs between maximising health and other objectives. The EHP TWG presented their draft package to District Health Officers and then the Ministry of Health management for approval. The whole process was facilitated by Ministry of Health economists.

The final agreed package costed $247 million per year and was predicted to avert 41.5 million DALYs if fully implemented. Like both previous packages, the cost of this package is more than the resources budgeted for it. However, it costs 31% less than the 2011 package ($362 million) and averts 92% as many DALYs. As such, it offers better value for money overall than its predecessor, implying significant progress towards a package that is more realistic and less aspirational.^17^ This also highlights that there are valuable health gains from expanding the budget for the package to its full cost. An example of the deliberative process undertaken by the Ministry of Health, alongside the analysis, was the decision not to include male circumcision in the final EHP despite the analysis showing it to be a ‘best buy’ intervention. It was judged that the type of demand-side constraints which would need to be overcome to increase the implementation levels would be too great and render the intervention not cost-effective.

The conditionalities of donors were considered in the process, particularly with respect to funding from the Global Fund and GAVI towards HIV and immunisation, respectively. After deliberation, the decision taken was to include many of the interventions funded by these organisations in the package, regardless of their cost-effectiveness, reflecting a lack of flexibility in health financing in Malawi and in the role of donors. The framework, however, provided a means to initiate conversation about the impact of a high proportion of earmarked funding within the health sector and the subsequent effect on population health.

The framework was augmented through further data collection on the additional criteria deemed important in package design within Malawi. Data in these fields were largely populated through expert elicitation. A benefit of the framework is its adaptable use in the policy-making environment. Additional data can be combined with the framework to the extent desired and possible. Within Malawi, quantitative data on other criteria considered for inclusion in the decision-making process (eg, financial risk protection) proved scarce, leading to the decision to focus primarily on health maximisation with other criteria for which data were elicitable from expert judgement considered within the deliberative process.

There were a number of limitations and challenges in using the analytic framework to revise the Malawi EHP. Initially there was limited understanding of opportunity cost, cost-effectiveness and budget constraint principles by some stakeholders. While the EHP TWG agreed on inclusion criteria, adhering to the implications of these choices was difficult in practice. In part, this was due to low total health expenditure per capita, $39, which suggested a much more restricted package than previous unaffordable packages. The historical vertical funding arrangements also meant that there was limited willingness by Heads of Departments and Programme Managers to consider disinvestment in their own interventions.

By applying the framework to data from Malawi, this study illustrates how metrics of value that reflect health opportunity costs can provide a principled and evidence-based support to decision-making processes. Specifically, they can quantify the health opportunity costs of constraints that inhibit delivering interventions fully; donor constraints on how funding is spent and the inclusion of objectives additional to improving population health.

Such analysis forms a critical part of package design. However, it also emphasises the important role of the decision-making process and how it interacts with analysis. As evidenced in the framework’s application in Malawi, that process needs to, for example, define the objectives of the package, deliberate on the relevance of the evidence provided by analysis and to make final decisions around what should (or should not) be included in the package.^27^ To ensure that it can be implemented, the package should also inform other health systems inputs and standards, such as treatment guidelines, essential medicines lists and payment or reimbursement mechanisms, which currently are not typically informed by such economic criteria. This can also inform broader questions such as the benefits of moving to a whole system approach to funding. For example, where funding is vertical and tied to one specific disease as is commonly the case, the health opportunity cost of this type of planning as opposed to a whole-system approach can be identified. The analysis also provides quantification of the health benefit of expanding the health sector budget and, therefore, clarifies trade-offs with other claims on public finance.

Inevitably the evidence available to conduct this analysis was limited in a number of respects. The interventions included in the analysis in this paper are those for which data were readily available on costs, health effects, the size of the patient population and actual levels of implementation. There were a number of interventions where some but not all of these data were available or were reported in ways that were not useful. There are also likely to be complementarities and interactions between interventions for which there is little evidence and have not been addressed, although the analysis can be extended to consider the cost and effects of different combinations of interventions. The analysis suggests that there are potentially substantial gains from investing in policies which reduce or remove constraints to implementation at the intervention level and across the HCS as a whole. However, additional evidence is needed about the cost and effects of specific policies and projects that could improve the implementation of high priority interventions.

## Conclusion

This study has illustrated the value of an analytic framework, founded on an understanding of the health opportunity costs of funding choices. It offers a transparent, principles-based approach to informing the content and scale of a HBP with existing resources, the value of expanding the HBP and the incremental reallocation of resources within the package. The paper has further shown that, even in the most evidence sparse of environments, available information can be marshalled and analysis presented in a way that empowers policy-makers and facilitates engagement of stakeholders in making explicit, accountable and evidence-based decisions on how limited resources can best be employed to improve population health. The Malawi case shows that the analytic framework is not prescriptive but rather a tool to guide decision-making that reflects the context in which they are made and which can be adapted and applied to different settings.
