# Supplementary material for: Supporting the development of a health benefits package in Malawi
Source: BMJ Glob Health. 2018 Apr 9;3(2):e000607. doi: 10.1136/bmjgh-2017-000607 (PMC5898345; doi:10.1136/bmjgh-2017-000607)
Supplement: Supplementary data [file bmjgh-2017-000607supp001.pdf]

Supplementary file 1. The costs of the conditionalities of donor funding: four example scenarios

The costs of the conditionalities of donor funding are illustrated through four scenarios assuming a level of affordability reflecting a health opportunity cost of $61 per DALY averted and an implied budget of $264.5 million:

1. Forcing interventions into the package
2. Conditional expansion of the package
3. Donor matched funding conditional on funding of a particular intervention
4. Donor matched funding without conditions

The cost of each arrangement is simply the difference in the aggregate health gains associated with a health maximising package and the package where the donor specified intervention is included and displaces one or more of the interventions in the health maximising package. Quantifying such costs provides a useful tool for communication and negotiation with donors.

The health opportunity costs of each scenario are reported in Table A1 where each scenario is compared against a health maximizing package. Scenarios 1-3 involve the donor requiring including in the package an intervention that would not otherwise have been included (i.e., as the package is based on health maximization and the intervention being forced in results in a negative net health benefit). This is illustrated using first line treatment for cervical cancer, which has a cost of $161,625 and averts 1 DALY.

Table S1. Comparing the implications of potential donor funding scenarios using first line cervical cancer screening

	Original package (i.e. all resources marshalled and allocated by the gov't to maximize population health)	Scenario 1	Scenario 2	Scenario 3	Scenario 4
		Forcing non cost-effective intervention into the package	Conditional expansion of the package	Matched conditional funding	Matched unconditional funding
Cost	$264,526,889	$264,526,889	$264,688,514	$264,607,701	$264,688,514
DALYs averted	49,451,522	49,425,932	49,425,933	49,464,317	49,477,113
Minimum health opportunity cost compared to a health maximising package at the same budget		25,590	21	12,795	none

The first scenario—forcing interventions into the package—involves a donor requiring that treatment for using first line treatment for cervical cancer is included in the package, although the donor is not increasing funding for the package. As such the budget remains the same, and the health opportunity cost is the health that would have been gained by whatever interventions must be removed from the package to fund first line treatment for cervical cancer. Removing the least cost effective intervention (i.e. those generating the least DALYs averted from their funding) in the package to fund first line treatment for cervical cancer tells us the minimum

health opportunity cost of forcing that intervention into the package: 25,590 DALYs averted. Any scenario where a non-cost-effective intervention is forced into the package at the expense of interventions that generate net health always results in a loss in the total health generated by the package.

Table S2. Opportunity cost of forcing first line treatment for cervical cancer into the package

	Cut		Forced in	Opportunity cost
#	48		65	
Program	Interventions focused on men who have sex with men		Cervical cancer (first line)	
	Total	Portion cut		
Cost	$1,255,745	$161,625	$161,625	
DALYs averted	198,831	25,591	1	25,590

The second scenario—conditional expansion of the package— also entails the inclusion of first line treatment for cervical cancer into the package, but rather than displacing other cost-effective interventions, the donor expands the package by $161,625 to $264.7 million conditional on that money being spent on first line treatment for cervical cancer. The health opportunity cost of doing this is the health that could have been generated by spending that additional money on the most cost effective interventions up to $161,625 that are not included in the package. In this instance, that results in an opportunity cost of 21 DALYs averted. Were first line treatment for cervical cancer the most cost-effective intervention outside the package this scenario would incur zero opportunity costs and would reflect an alignment of donor spending with government objectives (assuming an objective of maximizing health).

Table S3. Opportunity cost of conditionally expanding the package to include first line treatment for cervical cancer

	Next most CE program not currently in the package		Program forced in	Opportunity cost
#	49		65	
Program	High Cholesterol		Cervical cancer (first line)	
	Total	Portion that could be added		
Cost	$6,702,709	$161,625	$161,625	
DALYs averted	921	22	1	21

The third scenario—donor matched funding conditional on funding of a particular intervention—is a donor offer to provide additional funding as long as this is matched by the Malawian Ministry of Health and conditional on the funding going on a particular intervention. Instead of the donor funding first line treatment for cervical cancer in full, they are only willing to fund half (i.e., $80,812), requiring the remainder to come from the budget for the package. In order to free-up $80,812, the Ministry of Health will have to cut interventions and lose the health outcomes they achieve. Assuming again that the least cost-effective interventions are removed to release the $80,812, this results in 12,795 fewer DALYs averted than would be averted by the original package.

Table S4. Opportunity cost of donor matched funding conditional on funding first line treatment for cervical cancer

	Program cut		Program forced in	Opportunity cost
#	48		65	
Program	Interventions focused on men who have sex with men		Cervical cancer (first line)	
	Total	Portion cut		
Cost	$1,255,745	$80,812	$161,625	
DALYs averted	198,831	12,796	1	12,795

The fourth scenario—donor matched funding without conditions— is an offer to provide additional funding as long as this is matched but without a condition that the funding goes on a particular intervention. This provides a useful contrast to the third offer, as the additional matched funding is not conditional on the extra resources being devoted to a particular intervention not currently in the package, but instead can be used as considered appropriate by the Ministry of Health. Assuming again that the donor is again willing to provide $80,812 with matched funding from the budget for the package, given the Ministry's objective of maximising gains in population health, the absence of a condition means no existing interventions need to be removed from those funded by the original budget of $264.5 million. Indeed, the additional $80,812 from the donor can be used to add interventions to the package from those not currently funded, but these can be selected in order to maximise gains in health outcomes from the additional resources. On that basis, it would be possible to add six interventions. From the new effective budget of $264.7 million and with the addition of some treatment for high cholesterol, total DALYs averted would be 49.5 million. In contrast to the third scenario this represents a gain in population health overall, and would avert 12,797 more DALYs than scenario 3.
